# Development of computational models for microtesla-level magnetic brain scanning: a novel avenue for device development

**DOI:** 10.1186/s42490-022-00058-y

**Published:** 2022-01-24

**Authors:** Shane Shahrestani, Gabriel Zada, Yu-Chong Tai

**Affiliations:** 1 Department of Neurological Surgery, Keck School of Medicine, University of Southern California, CA Los Angeles, USA; 2 Department of Medical Engineering, California Institute of Technology, CA Pasadena, USA

**Keywords:** Brain, Neurology, Microtesla, Modeling, Computational, COMSOL multiphysics, Eddy current damping, Stroke, Hemorrhage

## Abstract

**Background:**

Detection of locally increased blood concentration and perfusion is critical for assessment of functional cortical activity as well as diagnosis of conditions such as intracerebral hemorrhage (ICH). Current paradigms for assessment of regional blood concentration in the brain rely on computed tomography (CT), magnetic resonance imaging (MRI), and perfusion blood oxygen level dependent functional magnetic resonance imaging (BOLD-fMRI).

**Results:**

In this study, we developed computational models to test the feasibility of novel magnetic sensors capable of detecting hemodynamic changes within the brain on a microtesla-level. We show that low-field magnetic sensors can accurately detect changes in magnetic flux density and eddy current damping signals resulting from increases in local blood concentration. These models predicted that blood volume changes as small as 1.26 mL may be resolved by the sensors, implying potential use for diagnosis of ICH and assessment of regional blood flow as a proxy for cerebral metabolism and neuronal activity. We then translated findings from our computational model to demonstrate feasibility of accurate detection of modeled ICH in a simulated human cadaver setting.

**Conclusions:**

Overall, microtesla-level magnetic scanning is feasible, safe, and has distinct advantages compared to current standards of care. Computational modeling may facilitate rapid prototype development and testing of novel medical devices with minimal risk to human participants prior to device construction and clinical trials.

## Background

Computational model generation represents an important tool for feasibility studies and study optimization, especially when designing and testing complex devices or methodologies. As such, computational models in neuroscience have gained a great deal of traction because they allow rapid prototyping and testing without the need for live human subjects, thereby reducing the risk of patient harm while advancing the device’s iterative designing process [1, 2]. In addition, computational models allow for theoretical testing of completely novel devices, highlighting yet unexplored avenues for device production [3, 4].

Our aim in this study is to develop computational models to evaluate the feasibility of microtesla-level magnetic brain scanning, including both detection of intracranial hemorrhage (ICH) and neural population activation. The current paradigm for ICH detection involves either magnetic resonance imaging (MRI) or computed tomography (CT) imaging, both of which involve expensive and non-portable equipment [5–7]. Similarly, one popular method of noninvasively quantifying neuronal population activity is functional magnetic resonance imaging (fMRI), which utilizes changes in blood flow and oxygenation (blood oxygen level dependent, or BOLD contrast) to predict neural activity [8]. ICH and increased neuronal activity share in common the feature of relative increases in local blood concentration compared to steady state, non-active brain, and wearable medical devices capable of detecting local changes in blood concentration may facilitate rapid prediction of hemorrhage or increases in brain activity.

Currently, there are no microtesla-level magnetic scanners being utilized within the medical field. Contemporary medical devices that leverage magnetic brain scanning include MRI and transcranial magnetic stimulation (TMS) devices, both of which operate on the order of several teslas. Here, we use finite element modeling (FEM) software to develop computational models for a wearable electromagnetic device capable of detecting hemodynamic changes within the brain. Using information from the computational model, we then develop and translate this device to a human cadaver setting to demonstrate its feasibility.

## Methods

### Proposed device physics

When carrying an alternating current (AC), solenoid coils generate time-varying magnetic fields which produce looping eddy currents within conductive materials according to Ohm’s Law. Non-destructive techniques based on eddy current damping (ECD) are widely used to test the presence, quantity, and integrity of various conductive materials, most commonly metals [9]. Eddy currents generated within the coil by a conductive material flow in the direction that decreases coil inductance and increases coil resistance, which may be used to quantify the degree of ECD produced. These ECD signals may be measured as a change in parallel resistance (R_p_) through the use of an electrical resonant circuit or LC tank, with a magnetic coil paired with a capacitor. When presented with a single or multi-dimensional space of magnetic flux density, the flux generated by eddy currents weakens the flux density within the entire field [10]. As such, it has been demonstrated that a correlation exists between magnetic flux density and ECD signals [11].

**Fig. 1:**
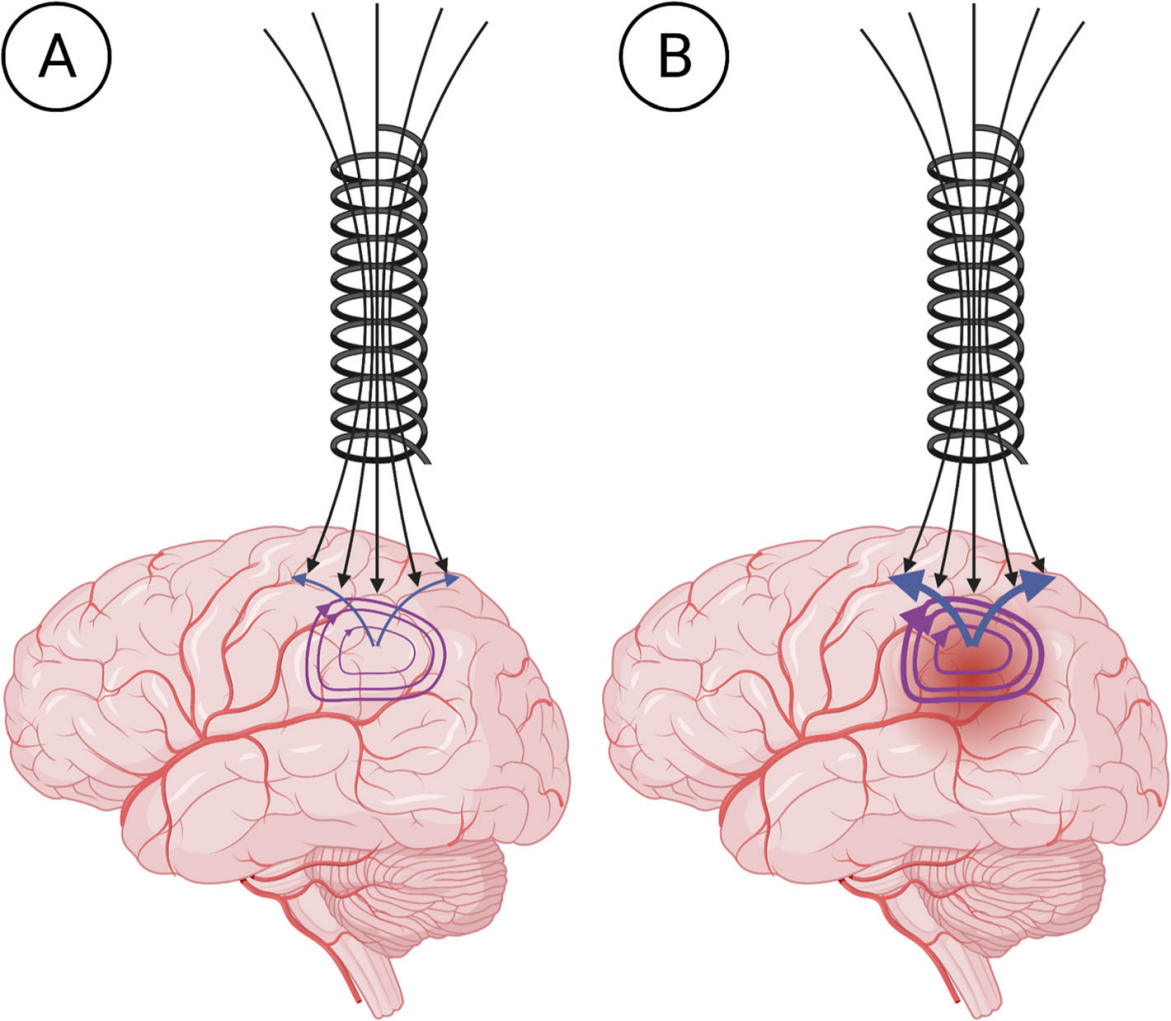
**A** When the coil is placed on the head, the primary magnetic field (black) generates looping eddy currents within the target (purple). These eddy currents generate a counteracting magnetic field (blue) which causes a measurable change in coil resistance, resonant frequency, and impedance. **B** When a more conductive hemorrhage is present, more eddy currents are generated, and a larger counteracting magnetic field is produced. Image created using Biorender.com

Because blood (0.65 S/m) is more electrically conductive than brain tissue (0.2 S/m) [12], we hypothesized that the presence of a more conductive material (blood) within a defined space in the brain (due to either ICH or increased local brain activity) may result in generation of a specific ECD signal, which is proportional to the magnitude, distance, and size of the blood-induced signal change according to a set of analytical solutions developed by Dodd and Deeds (Fig. 1) [13]. As such, a magnetic coil sensor capable of producing and measuring magnetic flux on the microtesla scale could detect ECD signals generated by local level intracranial hemodynamic changes.

### Finite element model

Using COMSOL Multiphysics version 5.5 (COMSOL Inc., Burlington, MA), we generated both three-dimensional (3D) and two-dimensional (2D) models of hemodynamic changes within a human head model, in addition to one-dimensional (1D) modeling of magnetic field decay as a function of distance. Text files of coordinate data from MRI head scanning were imported as an interpolation curve and organized in sectionwise format, and lofting of the closed curve allowed for the generation of a solid object roughly 20 cm in height and 10 cm in width [14, 15]. A novel material with a density of 2000 kg/m^3^, electrical conductivity of 0.4 S/m, relative permeability of 1, and relative permittivity of 568 was added to simulate the properties of a human head. An ellipsoid with dimensions of 0.035 m in the a-axis, 0.045 m in the b-axis, and 0.025 m in the c-axis was added within the head to simulate the brain. A novel material with a density of 1000 kg/m^3^, electrical conductivity of 0.2 S/m, relative permeability of 1, and relative permittivity of 827 was added to simulate the properties of the human brain. Lastly, a smaller ellipsoid with dimensions of 0.014 m in the a-axis, 0.018 m in the b-axis, and 0.010 m in the c-axis was added within the head to simulate an area of increased blood concentration. A novel material with a density of 1,060 kg/m^3^, electrical conductivity of 0.65 S/m, relative permeability of 1, and relative permittivity of 3,030 was added to simulate an area of increased blood concentration [16, 17]. Using the simplified formula for intracerebral hemorrhage volume, which estimates blood volume as half the product of all three ellipsoid semiaxes (ABC/2), we determined our small ellipsoid to represent a local increase of 1.26 mL of blood [18–21]. This volume was decided because previous studies have established that local brain activation may increase local blood concentration by approximately 1.5mL, and while adjusting the parameters of our COMSOL model, a volume of 1.26mL was the closest volume available to the 1.5mL target volume that we wanted to investigate [22].

Two scenarios were explored with reference to magnetic coil design: (1) a small handheld coil with 12.1 cm external diameter that could be moved across the head and (2) a larger coil with an external diameter of 27 cm that resembles a headband and is placed on the head circumferentially. Both scenarios shared the following parameters: peak current of 0.0027 amperes, 6 coil turns, coil width of 4.2 cm, and material properties including copper wire with a density of 8960 kg/m^3^, electrical conductivity of 5.998 × 10^7^ S/m, relative permeability of 1, and relative permittivity of 1. An infinite element with the material properties of air and modeled as a sphere was added encapsulating the entire head and sensor model.

### Mathematical models and computations

Ampere’s Law was implemented to calculate the electric current density as the curl of the magnetic field: $\nabla xH=J$(where $\nabla xH$ represents the curl of the magnetic field and *J* represents the electric current density). Electrical conductivity was modeled through Ohm’s Law: $J=\sigma E$(where *J* represents the electric current density, σ represents the material’s conductivity, and *E* represents the electric field). Lastly, magnetization ($B={\mu }_{0}{\mu }_{r}H$) and dielectric ($D={\epsilon }_{0}{\epsilon }_{r}E$) models were included, which utilized the relative permeability and permittivity values respectively to solve their corresponding equations. Testing frequency was set at 1 MHz and a stationary solver was implemented and finite elements included in the model utilized fine meshing with a total of 286,184 degrees of freedom in the small coil and 314,478 degrees of freedom in the larger coil. Several factors were considered when choosing 1 MHz. First, the LDC1101 inductance-to-digital converter chip used for experimental testing only operates at frequencies between 500 kHz and 10 MHz. Within this frequency range, only a handful of studies have thoroughly explored and published the electrical conductivities of biological tissues [12]. In many of these studies, conductivities within the 1 MHz region were thoroughly explored and validated. Lastly, we know that higher frequencies have shorter penetration into tissues and lower frequencies have higher penetration into tissues. After much thought, we settled to build the coil to operate at a frequency of 1 MHz to balance the pros and cons of sensor circuit limitations, available conductivity measurements from the literature, and penetration depth into the head.

### Cadaver model

A fresh, unfixed human body with no history of neurological or cerebrovascular disease was procured and laid in supine position. The quality of the brain was evaluated to ensure that it still retained properties of a live human brain. The head was lifted using pillows to about a 45-degree angle. We ensured that no metallic objects (i.e. metal table, head and neck implants) were in the range of detection of the coils. A head cap with 8 equidistant tubes was placed such that the most anterior tube crossed the forehead horizontally, and the most posterior tube crossed the nape of the neck horizontally. The neurosurgeon made an incision just above the right eyebrow to retract the skin and proceeded to drill a right supraorbital burr hole. The head was scanned by hand across all 8 rows with all three sensors to procure control brain data. Frozen uncoagulated porcine blood was left to thaw, and 60mL was withdrawn into a syringe and injected 7 cm deep into the brain parenchyma. The brain was scanned once more with all three sensors, this time with the bleed in place. Data was stored on a local computer for analysis. The cadavers were procured from the University of California, San Diego as part of the UC Anatomical Donation Program. Through this program, the deceased consented their bodies to research. Written consent was obtained.

## Results

### Magnetic field calculations

Using Biot-Savart’s Law, we can calculate the expected magnetic fields produced by both the small and large coils. These values can then be compared to those obtained through COMSOL modeling. For the smaller coil, the maximum magnetic field flux densities at the sensor (x=0) and 5 cm away from the sensor (x=5) are 0.1682µT and 0.0771µT respectively. For the larger coil, the maximum magnetic field flux densities at the sensor (x=0) and 5 cm away from the sensor (x=5) are 0.0754µT and 0.0622µT respectively.

### Small coil

#### 3D modeling

**Fig. 2:**
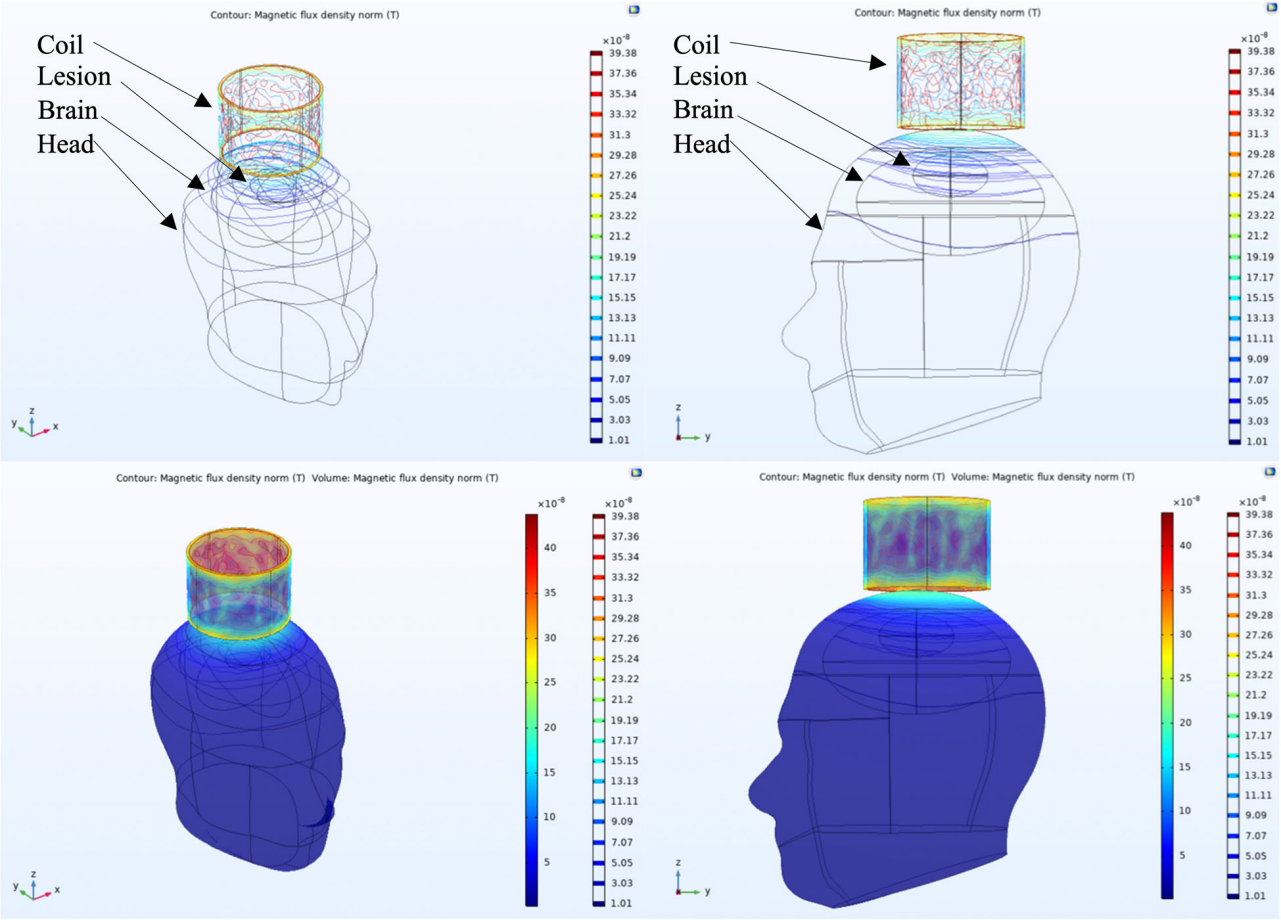
COMSOL multiphysics 3D simulations of the small coil on a human head model

We evaluated the magnetic flux density generated within a head model when a small solenoid coil was placed concentrically above an elliptical volume of increased blood concentration. Both contour and volume plots are shown in Fig. 2, where the microtesla-level magnetic fields generated by the sensor are sufficient to generate magnetic flux within the volume of blood, suggesting a measurable ECD signal is produced. In addition, the magnetic fields generated within the head model are comparable to those calculated via Biot-Savart’s Law, suggesting that the computational model accurately represents the expected physical phenomena.

#### 2D modeling

**Fig. 3:**
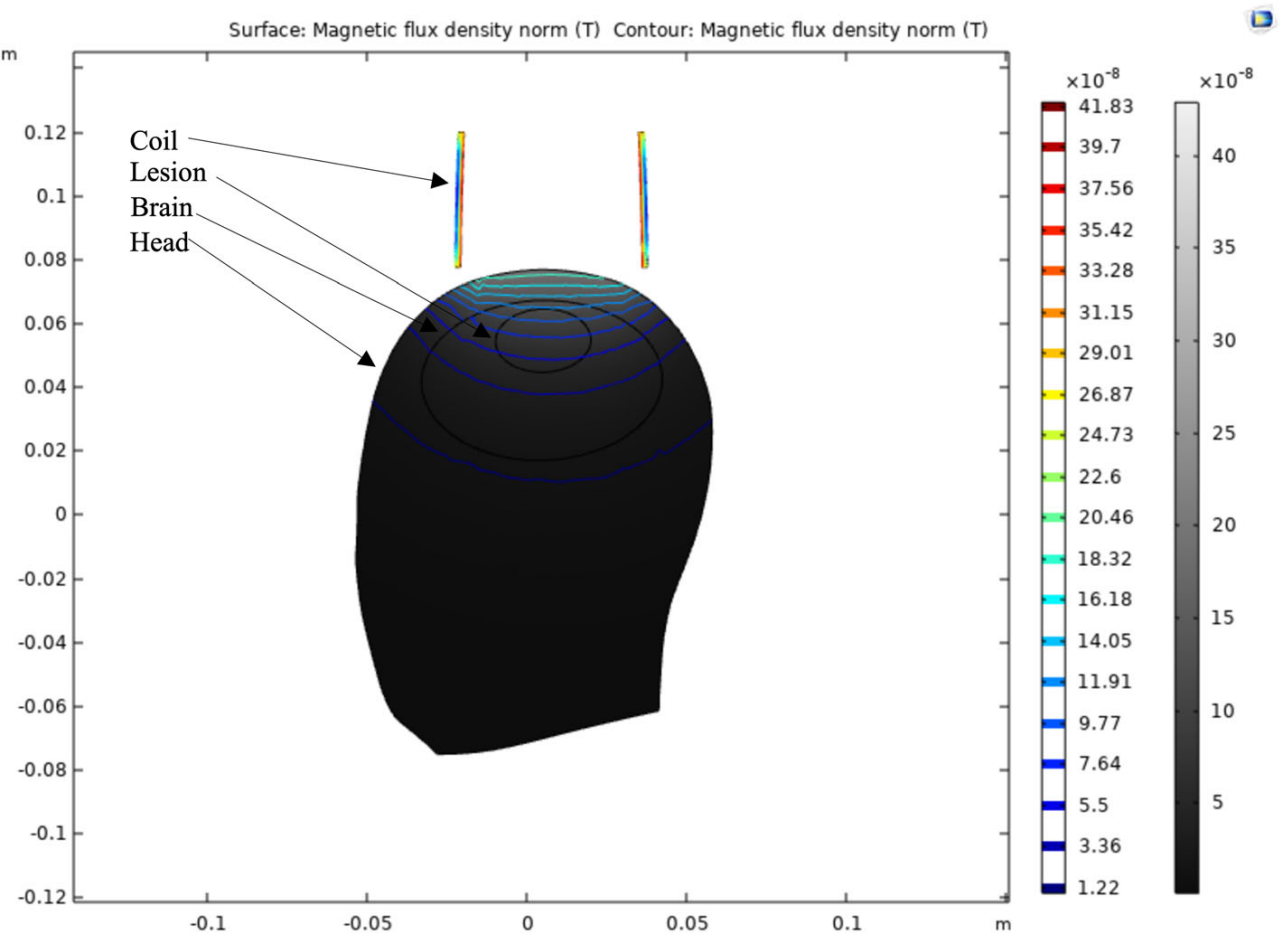
COMSOL multiphysics 2D simulation of the small coil on a human head model

The 3D model was projected on a 2D surface to better visualize the magnetic flux density profile generated by the small coil. As seen in Fig. 3, the magnetic flux decays as a function of distance, with the strongest magnetic flux being produced at the apex of the lesion. This finding suggests that microtesla magnetic sensors may be most sensitive to the hemodynamic changes closest to the sensor. Furthermore, the magnetic flux density suggests that the greatest magnetic flux is generated when both the coil and volume of blood are concentrically placed.

#### 1D modeling

**Fig. 4:**
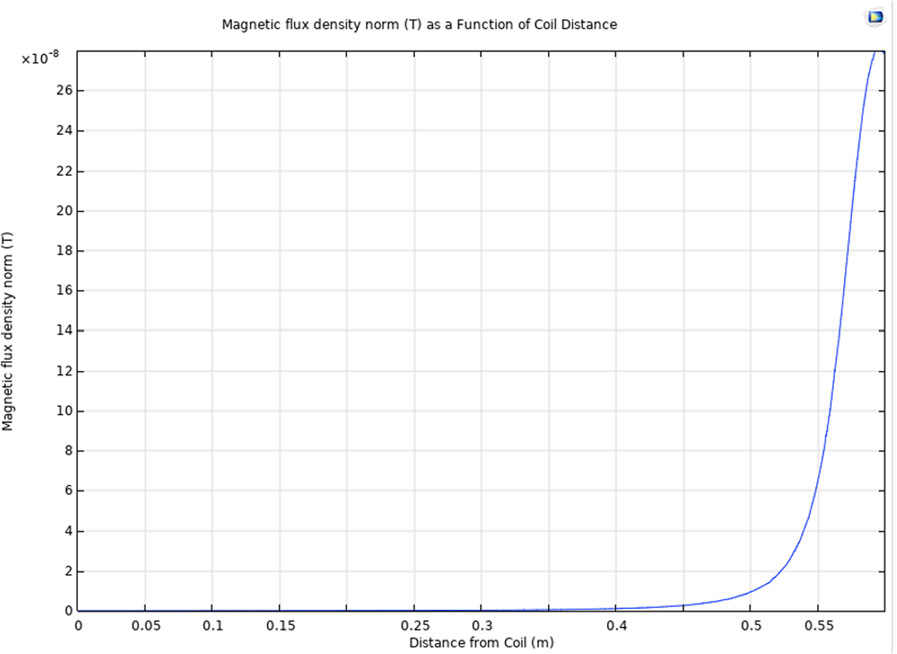
COMSOL multiphysics 1D simulation of the small coil on a human head model

To investigate the effects of distance on magnetic flux density, the head model was used to generate the 1D plot seen in Fig. 4. The bottom of the head is represented at x=0.0 m and the surface of the coil is represented at x=0.60 m, and the arc length used to generate this plot passed through the center of the head and coil model. As expected, the areas adjacent to the coil will produce the largest magnetic flux, which will decay as a function of distance. The ellipsoid representing the volume of blood was located roughly 5 cm away from the sensor (x=0.55) and from Fig. 4 we would expect a magnetic flux density of roughly 0.07µT. Calculations using Biot-Savart’s Law predicted a magnetic flux density of 0.0771µT at 5 cm, supporting the accuracy of our model.

### Large coil

#### 3D modeling

**Fig. 5:**
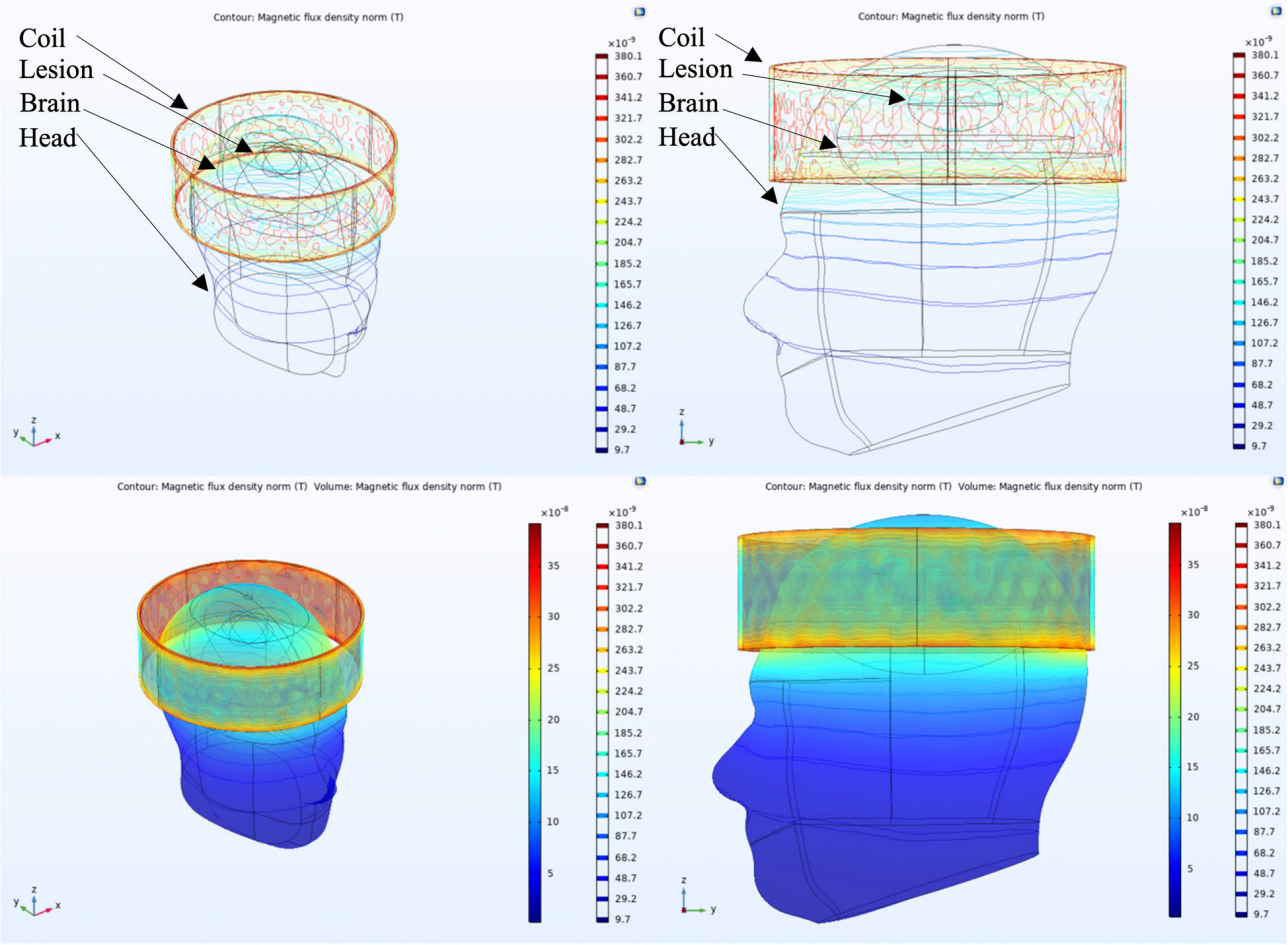
COMSOL multiphysics 3D simulations of the large coil on a human head model

The 3D model was generated with the larger coil circumferentially wrapping around the head model. Both contour and volume plots are shown in Fig. 5. The larger coil elicits a higher degree of magnetic flux within the head model. However, due to the high degree of head surface area scanned by the circumferential design, most of the magnetic flux density changes, and thus ECD signals, are produced by interactions with the tissue of the head and not the volume of hemorrhage.

#### 2D modeling

**Fig. 6:**
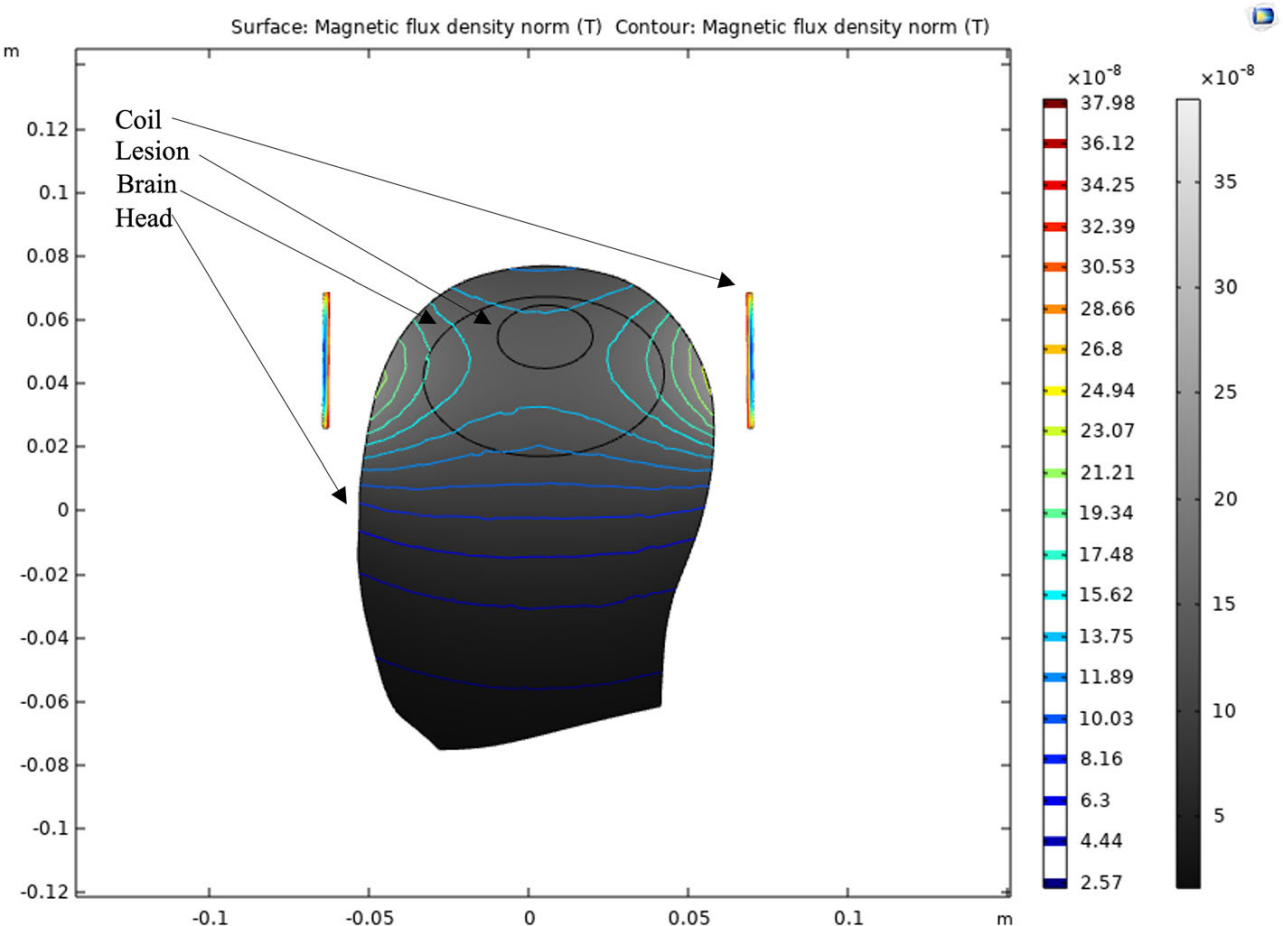
COMSOL multiphysics 2D simulation of the large coil on a human head model

To investigate the large surface area interactions introduced by a circumferential design, a 2D projection of the 3D head model was developed. A uniform magnetic field is seen in Fig. 6 throughout the entirety of the head model, with the greatest magnitude of magnetic flux at the edges closest to the coil. Further, little magnetic flux is seen within the elliptical blood model.

#### 1D modeling

**Fig. 7:**
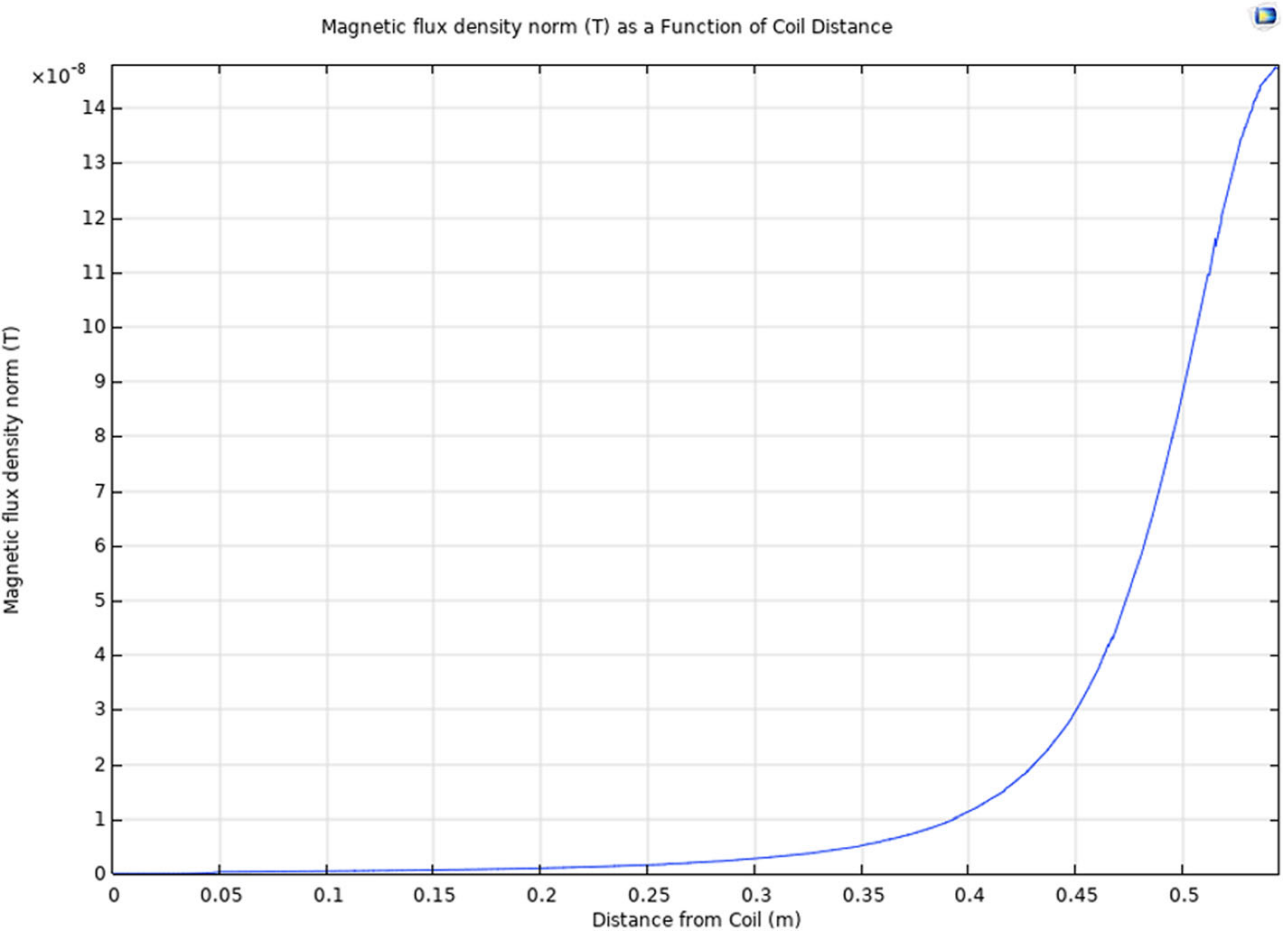
COMSOL multiphysics 1D simulation of the large coil on a human head model

To investigate the effects of distance on magnetic flux density, the head model was used to generate the 1D plot seen in Fig. 7. The bottom of the head is represented at x=0.0 m and the surface of the coil is represented at x=0.55 m, and the arc length used to generate this plot passed through the center of the head and coil model. Interestingly, the maximum magnetic flux density produced by the large coil was lower than that of the small coil, consistent with our Biot-Savart calculations.

### Sensitivity analysis

**Fig. 8:**
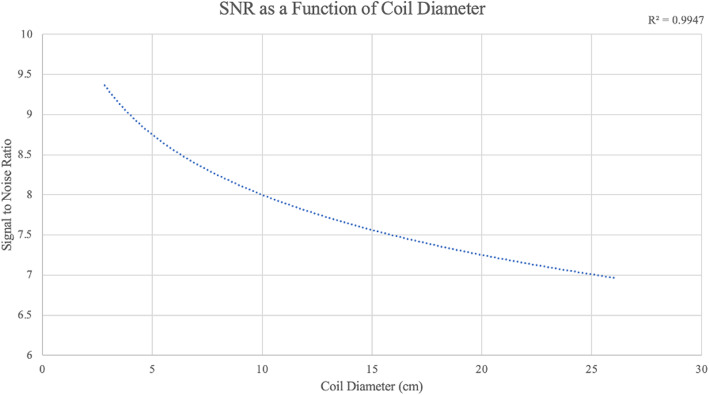
Signal-to-noise ratio (SNR) as a function of coil diameter

By experimentally constructing coils of various sizes, it was possible to calculate the signal-to-noise ratio (SNR) of each coil as a function of coil diameter (Fig. 8). This experimental finding suggested that the small coil may have a SNR of 7.8 while the large coil may have an SNR of 6.9. Additional sensitivity testing included the experimental calculation of coil noise-equivalent distance as a function of volume, which is defined as the distance at which SNR is equal to 1.00 for a given target volume. The noise-equivalent distance for each increase in 1mL was found to be 0.0018 cm, meaning that small targets require closer placement to the coil in order to be detected, while larger targets can be detected with the coil further away.

Comparing the predicted FEM magnetic fields to the calculated magnetic fields using Biot-Savart Law, we find that the predicted and calculated fields are nearly identical for both the small (predicted: 0.0707 µT, actual: 0.0771 µT) and large (predicted: 0.0816 µT, actual: 0.0622 µT) coils at a distance of 5 cm.

### Human cadaver experiments

**Fig. 9:**
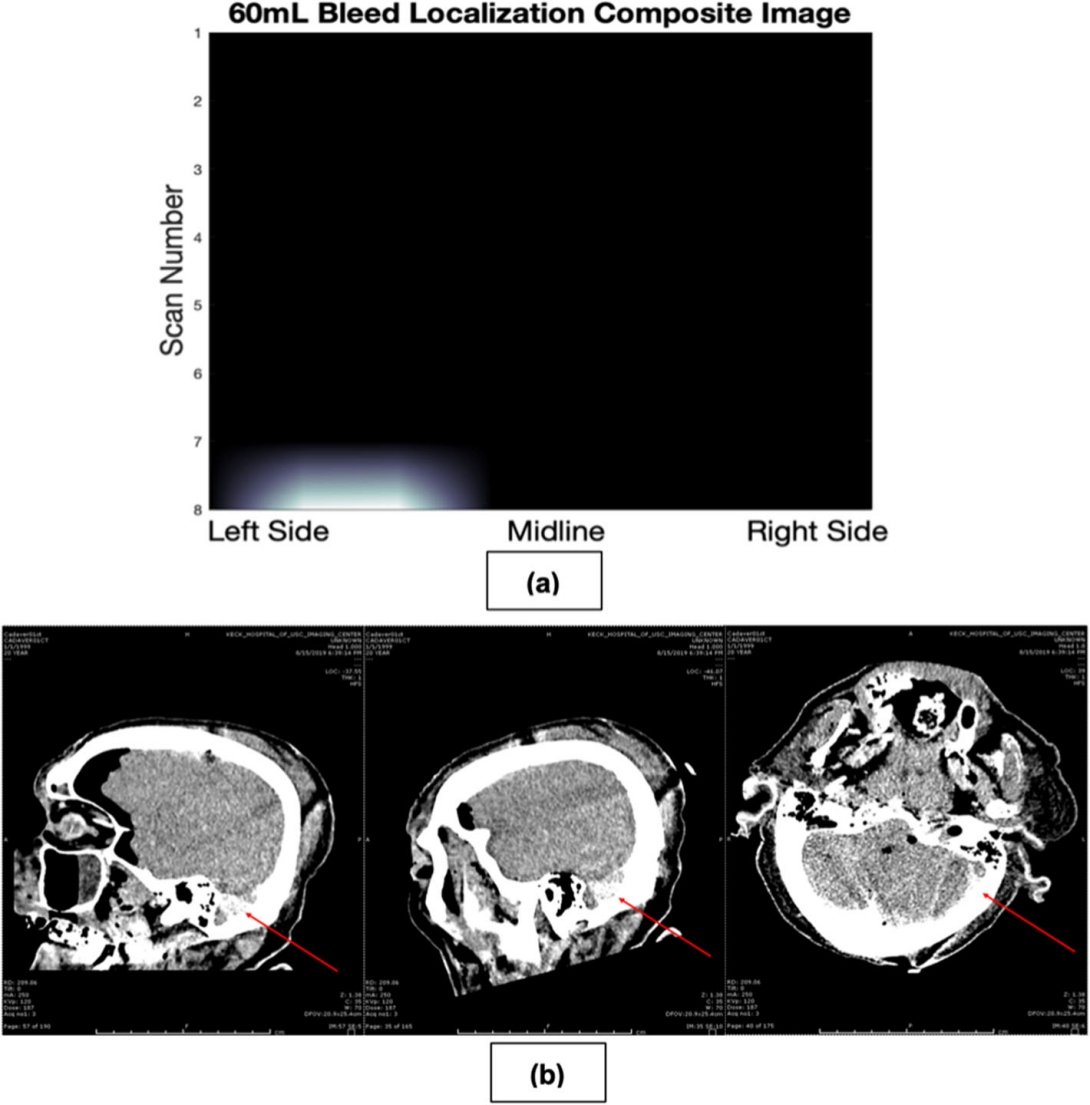
**a** Image produced by ECD sensor of 60mL porcine hemorrhage. **b** CT scanning of 60mL porcine hemorrhage corroborates findings from the ECD sensor, showing a left occipital ICH

From the computational models, it was clear that a smaller handheld sensor may reduce surface area interactions with the scalp and generate a larger magnetic flux density compared to the large coil. Similarly, the smaller coil had a higher SNR compared to the large coil, and thus, was more sensitive to signal rather than noise. Thus, we developed the small coil using copper Litz wire (46 AWG) and a commercial inductance-to-digital converting chip. In addition, ferrite shielding was added around the coil to minimize external magnetic interference. Pilot human cadaver studies (n=3) confirmed the feasibility of a sensor for the detection of intracranial blood through human skull bone and brain parenchyma. Frozen uncoagulated porcine blood was thawed and 60mL was injected directly through the burr hole, 7 cm deep into the brain parenchyma. Scanning the cadaveric head with the coil allowed for the production of a heatmap using continuous R_p_ measurements, which accurately predicted the location of the hemodynamic change (Fig. 9a). Interpreting the heatmap, we expected the modeled ICH to be located in the left occipital region, posterior to the left ear. Noncontrast CT imaging with bone and soft tissue (brain) windowing corroborated the presence of a hemorrhage in the left occipital region, the same located shown by the ECD sensor, and differentiated this finding from bone (Fig. 9b).

## Discussion

Our computational models suggest that microtesla-level static magnetic fields (SMFs) may be used to probe the brain for areas of increased blood concentration arising from either ICH or increased local brain activity. Excellent correlation was found between our FEM prediction models and actual experimental findings with the coil sensors. Neurovascular volume changes as small as 1.26mL may be resolved accurately as a local change in magnetic flux or through ECD signal processing, suggesting that portable magnetic coils or arrays of coils may be used as potential alternatives to MRI and fMRI for brain scanning for the purposes of detecting increased local blood concentrations. Using the optimal parameters determined through computational modeling, we developed a magnetic sensor and demonstrated its feasibility in a human cadaver setting.

The benefits of low-energy portable magnetic coils for brain scanning are quite clear in the realm of clinical neuroscience, neurology and neurosurgery. Current estimates suggest that the average time to treatment following ICH may be 2 h because MRI/CT imaging is required prior to treatment to rule out ischemic stroke, localize hemorrhage location, and plan for neurosurgical evacuation [23–25]. During this time, the rate of neuronal death is constant, contributing to the high rates of morbidity and mortality classically seen following hemorrhagic stroke [26]. Translational portable medical devices capable of accelerating the time to treatment in the field or at the patient bedside may potentially reduce the short and long-term complications following an ICH. The small magnetic field created by this sensor requires minimal current, which allows for device miniaturization and portability for testing outside the hospital.

In addition, the use of such a device for assessing neural activity on a regional level has tremendous potential in computational neuroscience. As previously stated, local brain activation may increase the local concentration of blood by approximately 1.5mL, which we show can be resolved by a microtesla-level magnetic field [22]. Increases in local blood flow following neuronal populational activation may be queried by an array of spatially targeted magnetic coils sensitive to magnetic flux density, which would theoretically produce a blood-dependent signal similar to that of BOLD-fMRI. While the use of magnetic coils would be much cheaper, require lower electromagnetic fields (EMFs), and reduce the risk of motion artifact because it could be directly attached to the head, it is still subject to many of the same limitations as BOLD-fMRI. Namely, both microtesla-level magnetic scanning and BOLD-fMRI represent indirect measurements of neuronal activity through the use of hemodynamic changes, which typically have a 1-2 s delay [27]. Lastly, the tradeoff seen in BOLD-fMRI when optimizing spatial and temporal resolution may also be seen in this device, and larger coils capable of probing deeper into the brain may lose sensitivity and temporal efficiency [28].

From our computational modeling, we can also determine that the use of microtesla-level magnetic brain scanning poses little to no physiological harm. MRI imaging and TMS devices typically operate on the order of several teslas and have achieved Food and Drugs Administration (FDA) approval with little potential for physical harm [29, 30]. As such, reduction of the SMF to the microtesla scale further reduces the risk of such a device. In addition, prior studies have established that EMFs in the millitesla range may interact with neuronal signaling, thus impairing walking in some experiments with locusts [31]. However, the threshold for physiological disturbances has been well studied and is believed to be set at 4mT for motor disturbances [31] and at 38mT for evoking miniature endplate potential (mepp) inhibition [32, 33]. Thus, the risk of adverse physiologic events or mepp disturbance is extremely low when using microtesla magnetic fields at 1 MHz.

### Limitations and future directions

While both the computational and cadaver models demonstrate the feasibility of such a device, our study is not without limitations. Firstly, interactions between the skin of the head and skull may dampen the magnetic flux and ECD signals analyzed by such a device, as seen in the computational models. However, the cadaver model demonstrated that the signal generated from the increase in local blood concentration may be greater than that of the skin and skull, and future work investigating potential sources of biological noise are warranted. Further, an inherent limitation to a magnetic sensor would be the requirement that all metallic objects are far from the scanning field to prevent the detection of high-conductivity objects. While we demonstrate the ability of such a sensor to detect an increased concentration of blood in a cadaver model, future live human testing is necessary to fully demonstrate the efficacy of such a device.

Lastly, while our computational models demonstrate the potential for detection of blood at lower volume limits as small as 1.26mL, limitations on current commercial inductance-to-digital converting (LDC) chips prevented this experimental finding during testing. Namely, the LDC 1101 chip utilized for this study had a non-ideal SNR which limited the detection of hemorrhage to volumes in the tens of milliliters due to internal noise within the commercial circuit. While our FEM analysis suggests that it is feasible to detect 1.26mL lesions, advancements in LDC architecture and signal processing are necessary to accurately detect volumes this small. Appropriate circuit optimization to achieve this feat may be an area of future investigation, although we demonstrate that it is theoretically feasible.

## Conclusions

Computational models can be developed to test the feasibility of novel medical devices and facilitate device development without risk to human participants. Models generated for microtesla-level devices show that they may be able to detect hemodynamic changes within the brain as a function of changes in magnetic flux density and ECD. Cadaver experimentation confirmed the feasibility demonstrated by our computational models, which facilitated device production by narrowing our parameter space. Such a device would greatly facilitate triage for hemorrhagic stroke and provide a novel method for functional neural population scanning. Further research and device development are required to translate these devices from the laboratory into the clinic.

## Data Availability

Data is available upon reasonable request. Please contact sshahres@caltech.edu.

## Abbreviations

ICH: Intracerebral hemorrhage
CT: Computed tomography
MRI: Magnetic resonance imaging
fMRI: Functional magnetic resonance imaging
BOLD: Blood oxygen level dependent
FEM: Finite element modeling
AC: Alternating current
ECD: Eddy current damping
R_p_: Parallel resistance
SMF: Static magnetic field
EMF: Electromagnetic field
TMS: Transcranial magnetic stimulation
FDA: Food and Drugs Administration
Mepp: Miniature endplate potential
